# Image quality of a portable X-ray device (Nomad Pro 2) compared to a wall-mounted device in intraoral radiography

**DOI:** 10.1007/s11282-020-00434-1

**Published:** 2020-03-30

**Authors:** Julia Nitschke, Lara Schorn, Henrik Holtmann, Uwe Zeller, Jörg Handschel, David Sonntag, Julian Lommen

**Affiliations:** 1 Department of Neurosurgery, Heinrich-Heine-University, Moorenstr. 5, 40225 Duesseldorf, Germany; 2 Department of Oral-, Maxillo- and Plastic Facial Surgery, Heinrich-Heine-University, Moorenstr. 5, 40225 Duesseldorf, Germany; 3 Department of Oral and Maxillofacial Surgery, Malteser Clinic St. Johannes, Johannisstraße 21, 47198 Duisburg, Germany; 4 Consulting Engineer for the Study Design and Regulatory Aspects, Rissegger Steige 139, 88400 Biberach, Germany; 5 Department of Oral and Maxillofacial Surgery, Kaiserteich Medical Center, Reichstr. 59, 40217 Duesseldorf, Germany; 6 Medical School, Heinrich-Heine University, Universitätsstr. 1, 40225 Duesseldorf, Germany; 7 Department of Conservative Dentistry, Parodontology and Endodontology, Heinrich Heine-University, Moorenstr. 5, Duesseldorf, Germany

**Keywords:** Handheld, Device, Dental, Radiology, X-ray

## Abstract

**Objectives:**

The aim of this study was to determine whether a handheld (HH) X-ray device (Nomad Pro 2) is capable of producing equivalent or even superior X-ray image quality in comparison to a wall-mounted (WM) dental X-ray unit (Heliodent Plus) on the basis of objectifiable image quality parameters.

**Methods:**

Anatomical, radiological and biological dental X-ray image quality parameters of a handheld dental X-ray device (Nomad Pro 2, Kavo Kerr, Biberach, Germany) were compared to a standard wall-mounted dental X-ray unit (Heliodent Plus, Sirona Dental Systems, Bensheim, Germany) using a maxillofacial phantom. In addition, the effect of different operators (dentists, dental students, dental assistants) on the dental X-ray image quality was measured.

**Results:**

HH and WM devices showed comparable image quality for anterior teeth, premolars, molars and bitewing images. During the two-month investigational period, the radiation exposure level for the operator of the Nomad Pro 2 was 0.1 mSv for 203 images. Dentists as the highest trained personnel enrolled in the study achieved better image quality with the Nomad Pro 2 as compared to dental students and dental assistants, especially in the molar region.

**Conclusions:**

A HH device delivers a comparable image quality to a WM device. In addition, there seem to be short learning curves with regard to image acquisition when using a handheld device, which is further minimised by the previous training of the operating personnel. HH dental X-ray devices, such as the Nomad Pro 2 are a promising adjunct for dental radiology in cases where WM units are of limited practicability.

## Introduction

In contemporary clinical dentistry, the use of X-ray images fundamentally increases diagnostic and therapeutic quality. Accordingly, orthopantomograms and dental X-rays are the preferred radiographical examinations not only in cases of dental trauma, but also in conservative dentistry and prosthodontics. Therefore, dental diagnostic imaging remains one of the most commonly performed radiological procedures worldwide [1]. This might have an impact on the lifetime radiation exposure of the general population, promoting malignancies, such as thyroid cancer [2].

Today, intraoral dental X-ray units are typically wall mounted (WM). Such configuration or design goes back to the original technical constraint in which a high voltage cable, attached to the so-called scissor arm, is needed to connect the X-ray tube assembly to the high voltage generator. This basic design of using a scissor type arm has not been changed once it became feasible to embed the high voltage generation into the X-ray tube head [3]. This WM design allows for positioning of the dental X-ray tube and has been the base of teaching intra-oral X-ray acquisition for multiple decades. The obvious disadvantage is the immobility of such devices, unless being fixed on a wheeled stand [4]. In cases of immobile, severely ill or disabled patients, it has proven difficult, if not impossible, to obtain dental X-ray images. This often leads to the problem of insufficient diagnostic statements and might lead to incorrect therapeutic decisions. Recently, handheld (HH) dental X-ray devices, such as the Nomad Pro 2 (Kavo Kerr, Biberach, Germany), were introduced as an alternative to the conventional WM units [5]. These devices claim to offer a convenient and portable size and are supposed to be easily operated even under difficult conditions, such as bedside dental X-ray imaging in hospitals and nursing homes, while offering equivalent operator doses to standard WM units [6]. This new class of devices is, in some regions, covered by regulatory guidance [7].

The guidelines of the German Federal Medical Association for quality assurance in radio diagnostics require dental X-ray images to fully capture the examined tooth from crown to apex, the periodontal bony structure as well as the border of the inner alveolar cortex to the periodontal space [8]. Furthermore, standards for dental X-ray diagnostics authored by the German Federal Association of Dentists dictate imaging voltages of ≥ 60 kilovolts (kV), a resolution of ≥ 5 line pairs per millimeter (mm) as well as a distance between the focus and the end of the X-ray tube of ≥ 200 mm [9]. The average radiation effective dose determined for analogue dental X-ray images is approximately 0.005 millisieverts (mSv) and 0.003 mSv for digital dental X-ray imaging. Whether portable handheld X-ray devices are capable of providing all of the required image quality parameters on a reproducible and safe basis is currently being debated, limiting the use of handheld X-ray devices in Western Europe [10].

Hence, the aim of this study was to determine whether the HH X-ray device Nomad Pro 2 (Kavo Kerr, Biberach, Germany) is capable of producing equivalent X-ray image quality compared to the WM dental X-ray unit Heliodent Plus (Sirona Dental Systems, Bensheim, Germany) on the basis of objectifiable image quality parameters using a real tooth phantom. Furthermore, dependence of different operators (dentists, dental students and dental assistants) on the X-ray image quality parameters of the Nomad Pro 2 and the Heliodent Plus was assessed.

## Methods and materials

In this study, the HH Nomad Pro 2 (Kavo Kerr, Biberach, Germany) was used to conduct fully hand-guided dental X-ray images by means of a digital dental X-ray sensor (GXS 700, size 1 and 2, Gendex, Kavo Kerr, Biberach, Germany). X-ray image quality parameters (according to the current statutes of the German Dental Association; [9]) of the Nomad Pro 2 were then compared to a typical WM X-ray unit (Heliodent Plus, Sirona Dental Systems, Bensheim, Germany) with the same digital dental X-ray sensors (GXS 700, size 1 and 2, Gendex, Kavo Kerr, Biberach, Germany). Quality parameters were distortion, level of detail, image size, overlay, resolution, radiation field, and technical parameters, such as distance from tube end to focus and receiver dose (see Table 1).

**Table 1:** Compared variables between the Nomad Pro 2 and the Heliodent Plus (according to [9]; mandatory criteria for bite wing images^a^)

Variable	Dichotomic scale
Complete imaging of diagnostically relevant structures with as little geometrical distortion as possible^a^	Yes/no
Display of the periodontal gap and the lamina dura	Yes/no
Display of the periradicular bone structure	Yes/no
Display of the alveolar crest	Yes/no
Display of the pulp cavity and root canals	Yes/no
Display of the entire dental crown including the proximal region^a^	Yes/no
Overlay effect on relevant structures	Yes/no
Receptacle voltage ≥ 60 kilovolts (kV)^a^	Yes/no
Nominal focal spot value ≤ 1.5^a^	Yes/no
Size of the radiation field at the end of the tube ≤ 60 mm diameter^a^	Yes/no
Blanking to film format, if possible and reasonable	Yes/no
Distance from tube end to focus ≥ 200 mm^a^	Yes/no
Image receiver dose ≤ 200 μGy^a^	Yes/no
Spatial resolution: ≥ 5 line pairs/mm [11]^a^	Yes/no

**Fig. 1:**
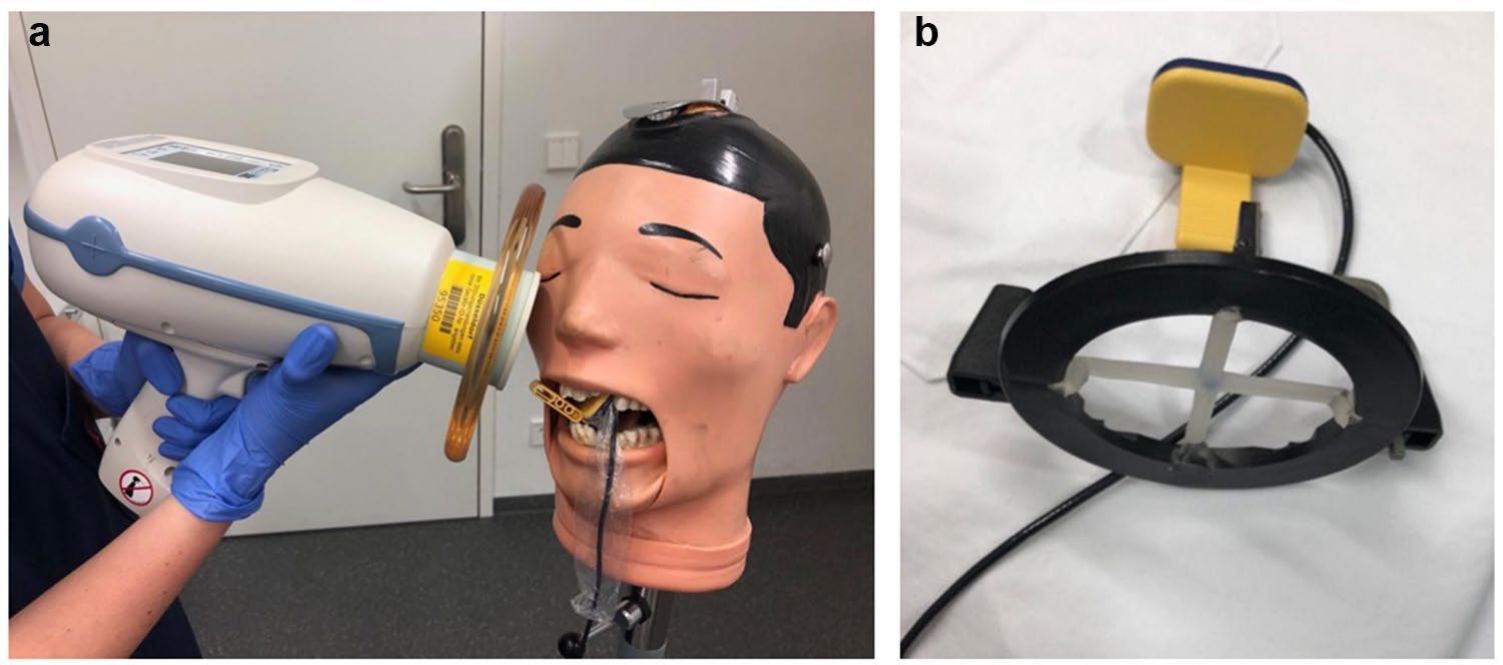
Experimental setup: **a** placed sensor inside the real tooth phantom and Nomad Pro 2 before image triggering. **b** Gendex digital X-ray sensor with positioning device

In order to record these X-ray image quality parameters, dental X-ray images as well as bite wing images (mandatory criteria for bite wing images are marked with an asterisk in Table 1) of the anterior teeth, premolars and molars of the left and right maxilla and mandible were conducted with a real tooth phantom (DXTTR mannequin, Densply, Ontario, USA). All shots were taken using a positioning ring with a metal rod (Fig. 1b). Images were taken holding the HH device first (Fig. 1a). Afterwards, all images were taken again using the WM device. Each image was positioned and shot individually.

All examinations were carried out and controlled by the study leader (specialist for oral and maxillofacial surgery) and a medical student in her 5th year of study.

**Fig. 2:**
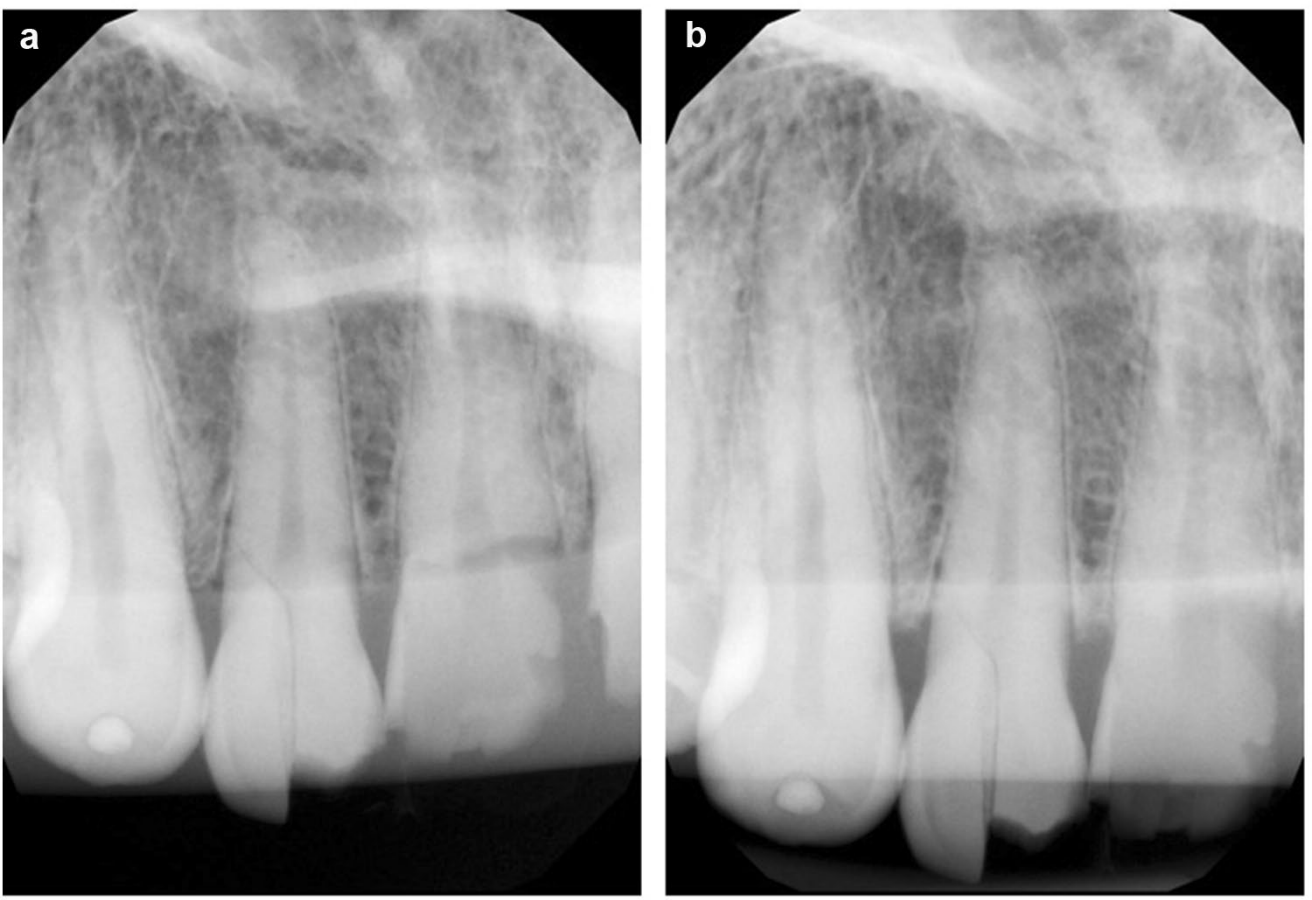
Radiological images of the maxillary anterior teeth taken by the HH Nomad Pro 2 (**a**) and the WM Heliodent Plus (**b**)

Furthermore, three dental students (having taken dental X-ray images supervised for 2 years), three dental assistants (> 5 years of work experience) and three dentists (> 5 years of work experience) were instructed to use the Nomad Pro 2 and conduct X-ray images with the real tooth phantom. The dental students had to be in their clinical study phase, the dental assistants had to have completed their vocational training and the dentists had to have two years of experience on the job after graduation. Everyone received a 20-min introduction and training with the HH device before the X-ray images were taken. The following images had to be taken independently: Ten dental X-ray images of (1) the anterior teeth (Fig. 2), (2) ten of the premolars and (3) ten of the molars of the maxilla *and* mandible in each quadrant (*n* = 120) Additionally, ten bite wing images (4) (of molars/premolars) were taken on each side with the Nomad Pro 2 (*n* = 20, in total *n* = 140) (Table 2). They were compared and analysed using the image quality parameters described above as well (Table 1).

**Table 2:** Methodical approach to take X-ray images

Conducted series of X-ray images
10 dental X-ray images of (1) the anterior teeth, (2) premolars, (3) molars and (4) bite wing images of the maxilla and mandible with the Nomad Pro 2 and the Gendex Sensor (total = 140 X-ray exposures)
10 dental X-ray images of (1) the anterior teeth, (2) premolars, (3) molars and (4) bite wing images of the maxilla and mandible with the Heliodent Plus and the Gendex Sensor (total = 140 X-ray exposures)
After every X-ray image Nomad Pro 2 and Heliodent Plus were newly repositioned from starting position
Calibration of the Nomad Pro 2 and Heliodent Plus with a dosimeter (Thermo Eberline ESM, ThermoFisher Scientific, Waltham, MA, USA) to obtain equivalent radiation doses at the sensor: Handheld Nomad Pro 2: 60 kV, 0.12 s, 2.5 mA Wall-mounted unit Heliodent Plus: 60 kV, 0.06 s, 7 Ma

The real tooth phantom used in this study has been used for approximately 20 years in numerous scientific international studies and has been validated to physiologically represent the dentoalveolar anatomy [11, 12].

### Viewing conditions

Only an accepted and constancy checked diagnostic monitor approved according to German standards (DIN standard 6868-157) was used for the examination and diagnosis of the generated dental film images of both the HH and the WM system.

### Image receptor alignment and recording space

Sensor alignment has been identical for both WM and HH device generated images. The prescribed and approved sensor holders for the GXS 700-sensor, size 1 and 2 (Gendex, Kavo Kerr, Biberach, Germany) were used. The experimental setup is shown as an example in Fig. 1. The images were taken in the approved X-ray room, in which the remaining standard dental X-ray units are also located.

### Radiation protection aspects

Throughout this study, a shared film-based X-ray dosimeter (whole-body dosimeter; radiation type: X-ray s and gamma rays; measurement: depth person dose; measuring range 0.1 mSv–1 Sv, 13 keV–1.4 MeV) was worn by all investigating personnel in order to measure released radiation (carried at chest height on an X-ray apron approved for dental radiology) for each taken image (separate dosimeters for HH and WM devices). Between active image acquisition it was stored in a radiation protected room (image release chamber for dental X-ray devices). After the acquisition sequence, the X-ray dosimeter was read according to the routine praxis of radiation dose monitoring once a month by local material testing office (the reading of the dosimeter was corrected for natural, background dose).

### Ethics approval and trial registration

This study was approved by the Ethics Committee of the University of Duesseldorf (Study number: 2018-162-KFogU). Trial registration of prospective trials: Central Study Register of Duesseldorf University Hospital, Registration-ID: 2018064716. Additionally, this study was approved by the district government of North Rhine-Westphalia due to the use of X-ray radiation (approval 772/18).

### Statistical methods

A statistical power analysis was used to determine a significant sample size (G*Power open source software [13]. Data analysis was performed using IBM SPSS statistics for Mac version 26 (SPSS Inc., Chicago, IL USA) and Microsoft Excel for Mac version 16.16.3 (Microsoft, Redmond, WA, USA). Frequency distributions for the analyses of image quality parameters were examined with the help of cross-tabs (Pearson’s Chi-square test). Due to skewed/abnormal distribution, nonparametric tests were used, additionally, to compare the image quality parameters between the HH Nomad Pro 2 and the WM Heliodent Plus (Mann–Whitney *U*, Kruskal–Wallis *H*). The level of significance was set to *p* ≤ 0.05. Values of *p* ≤ 0.01 were considered highly significant.

## Results

### Percentage of cases/pictures with all mandatory diagnostic criteria fulfilled

Analysis of the image quality parameters between the HH Nomad Pro 2 and the WM Heliodent Plus of all dental regions taken together (anterior teeth, premolars, molars) show an insignificant trend in favour of the Nomad Pro 2 in the maxilla (40.0–33.3%; *p* > 0.05) and mandible (45.0–31.7%; *p* > 0.05). Combining the values of maxilla and mandible, *p* value is also insignificant with a trend towards Nomad Pro 2 (36.4–27.9%; *p* > 0.05).

Individual analysis of the image quality parameters of the respective dental regions reveals a significant difference between the Nomad Pro 2 and the Heliodent Plus in favour of the Nomad Pro 2 for the front teeth of the maxilla (55–15%, *p* = 0.005) and the front teeth of the mandible and the maxilla together (30.0–7.5%, *p* = 0.034). No significant differences in the image quality parameters between the Nomad Pro 2 and the Heliodent Plus were found in the analysis of the bite wing X-ray images (0%; *p* > 0.05).

### Percentage of criteria fulfilled absolutely

Due to the skewed distribution of the percentage of fulfilled criteria, nonparametric tests were used to compare the percentage of fulfilled criteria between the Nomad Pro 2 and the Heliodent Plus. These data show a significant difference in favour of the Nomad Pro 2 (*p* = 0.044) as compared to the Heliodent Plus for total taken images. No significant differences could be found within the specific dental regions (anterior teeth, premolars, molars). On the contrary, analysis of the bite wing X-ray images showed a significant difference between the Nomad Pro 2 and the Heliodent Plus in favour of the Nomad Pro 2 (*p* < 0.001).

### X-ray image quality differences of the Nomad Pro 2 handled by different occupational groups

When the HH Nomad Pro 2 was operated by the three dentists included in this study image quality parameters were significantly better in the maxillary molar region (*p* = 0.047) and the mandibular premolar region (*p* = 0.028) compared to the WM Heliodent Plus. Furthermore, the Nomad Pro 2 showed significantly better image quality parameters in the entire mandible (*p* = 0.037) and all premolar regions of the maxilla and mandible (*p* = 0.014). In all other regions examined, equivalent image quality parameters of the Nomad Pro 2 and the Heliodent Plus were demonstrated.

When the HH Nomad Pro 2 was operated by the dental students, statistically significant differences (*p* = 0.046) between the Nomad Pro 2 and the WM Heliodent Plus were exclusively seen for all molar regions in the maxilla and mandible in favour of the Nomad Pro 2. There were no significant differences in image quality parameters for images of the anterior teeth or premolars.

Dental assistants operating the HH Nomad Pro 2 showed significant differences (*p* = 0.033) in favour of the Nomad Pro 2 solely for the images of the entire mandible (anterior teeth, premolars and molars). In the maxilla, there were no differences between the Nomad Pro 2 and the WM Heliodent Plus.

Comparing the outcomes of image quality parameters of the Nomad Pro 2 for dentists, dental students and dental assistants only the group of dentists showed a significant advantage (*p* = 0.050) of the Nomad Pro 2 in the premolar region as compared to dental students and dental assistants. Comparing outcomes for dentists, dental students and dental assistants operating the wall-mounted Heliodent Plus, dentists and dental assistants showed a significant advantage (*p* = 0.050) over dental students in taking X-ray images of the premolar region in the maxilla. In all other areas, no significant differences were observed.

### Total amount of X-ray radiation released in the course of this study

Within the study period between 01.08.2018 and 30.09.2018, released (stray) radiation dose for all operating participants was 0.1 mSv as measured by the used dosimeter all together for all HH conducted images (*n* = 203). There was no radiation dose detected for WM devices.

## Discussion

The results of the present study indicate an equivalent X-ray image quality when using a HH Nomad Pro 2 device in comparison to the WM X-ray unit Heliodent Plus in X-ray images of the premolars, molars and bitewing images. Since the same digital sensors and identical examination conditions have been used for the Nomad Pro 2 and the Heliodent Plus to acquire comparable results these findings are in accordance with a study by Ulusu et al., who found equivalent image quality of handheld devices on digital and conventional bitewing radiographs [14]. The Nomad Pro 2 even surpassed image quality parameters of the Heliodent Plus in dental X-ray images of the anterior/front teeth (counting only images fulfilling all necessary criteria) as well as the percentage of fulfilled criteria for all dental regions together and the bite wings in particular. A reason could be difficult alignment of the wall-mounted tube central ray perpendicular to the image receptor. The finding that especially dental X-ray images of the anterior teeth region showed superior image quality of the Nomad Pro 2 compared to the Heliodent Plus supports the hypothesis that angulation and adequate positioning of the handheld Nomad Pro 2 might be easier than the wall-mounted X-ray tube head of the Heliodent Plus. Pittayapat et al., also found that the image quality of handheld dental X-ray devices was adequate for forensic use [15]. In our study, we found no evidence that the dental X-ray image quality of the Nomad Pro 2 was lower compared to the wall-mounted unit. It might, therefore, be useful when treating immobile, disabled or severely ill patients. In an ageing, less mobile population the need for portable devices will on the long run be necessary to provide adequate diagnostic and therapeutic options. The use of a portable device, such as the Nomad Pro 2 could be imaginable in nursing homes or intensive care units.

Furthermore, the “as low as reasonably achievable; ALARA” principle could be regarded as higher for the Nomad Pro 2, compared to a wall-mounted dental X-ray unit considering radiation exposure for the wall-mounted unit being between 0 and 0.1 mSv (yearly full-body effective dose) taken from a radiation protected area at a distance or behind shielding [7, 16]. However, with a radiation dose of 0.1 mSv for about 2 months and 203 taken images, still corrected for natural background radiation and including potential use errors (e.g., one of the users standing unprotected or too close to the phantom while taking HH images) for all personnel enrolled in the study together the operator exposure levels remain below recommended levels of the Ionizing Radiation Regulations of 1999 as described in other studies [16] and the measured scattered radiation values of dental HH X-ray units of a current study, which indicates limit values of 1.32–2.55 mSV annually [7]. A study by Rottke et al. [17] showed similar results. The radiation was measured using a film dosimeter as whole-body dosimetry. The dosimeter was protected by shielding. Scatter above and below the Nomad shield can reach the operator and, in these areas, the reduction in scatter intensity due to the inverse square law is offset by the absence of shielding. When using a WM device, the operator took the image from a 2 m distance and behind shielding, therefore, no radiation could be detected while using WM devices. Training of operating personnel might influence radiation when using a HH device. In order for the operator's body to be parallel and behind the scatter shield, the head of the patient must be tipped down for anterior maxillary imaging and up for anterior mandibular imaging. When this step is not taken, the required downward angle of maxillary imaging results in increased operator abdominal/gonadal exposure while upward angulation of mandibular imaging results in increased exposure of the clinician’s thyroid gland from patient backscatter.

It was stated in earlier studies that training of the operator also has a higher effect on the precision of aim than the type of device (HH vs. WM) [6]. In part, we support this statement since dentists as the highest trained personnel included in this study achieved better results with the Nomad Pro 2 compared to dental students and dental assistants, especially in the molar and premolar region. No differences, however, were found for anterior teeth, presumably because sensor and handheld device can be positioned more easily in this area. On the whole, our results in this respect are similar to those of a recent study by Hoogeveen et al., who do not see any difference in target precision between HH and WM devices. In contrast to our study, however, they only used dental students as operators [5]. Collectively, additional studies with more personnel in different stages of training need to be conducted to test for operator-dependent differences in dental X-ray image quality.

## Conclusion

The Nomad Pro 2 delivers an image quality that is at least as good as that of a WM device. In addition, there seems to be an easy learning curve with regard to the HH image acquisition procedure. The use of HH dental X-ray devices might be advantageous in daily clinical routines.

## References

[1] Han GS, Cheng JG, Li G, Ma XC (2013). Shielding effect of thyroid collar for digital panoramic radiography. Dento Maxillo Fac Radiol.

[2] Crane GD, Abbott PV (2016). Radiation shielding in dentistry: an update. Aust Dent J.

[3] Scheuzel P (1995). Wilhelm Conrad Röntgen—Unsichtbares wird sichtbar.

[4] Ritter AG. Röntgentechnik mit dem Ritter Röntgen-Apperat Teil 1.: RITTER Karlsruhe-Durlach; 1950;1;55–78.

[5] Hoogeveen RC, Meertens BR, Berkhout E (2019). Precision of aiming with a portable X-ray device (Nomad Pro 2) compared to a wall-mounted device in intraoral radiography. Dento Maxillo Fac Radiol..

[6] Gray JE, Bailey ED, Ludlow JB (2012). Dental staff doses with handheld dental intraoral X-ray units. Health Phys.

[7] Smith R, Tremblay R, Wardlaw GM (2019). Evaluation of stray radiation to the operator for five hand-held dental X-ray devices. Dento Maxillo Fac Radiol..

[8] Leitlinie der Bundesärztekammer zur Qualitätssicherung in der Röntgendiagnostik, Qualitätskriterien röntgendiagnostischer Untersuchungen. 2017. https://www.bundesaerztekammer.de/fileadmin/user_upload/downloads/LeitRoentgen2008Korr2.pdf.

[9] Firetto MC, Abbinante A, Barbato E (2019). National guidelines for dental diagnostic imaging in the developmental age. Radiol med..

[10] Pittayapat P, Oliveira-Santos C, Thevissen P, Michielsen K, Bergans N, Willems G (2010). Image quality assessment and medical physics evaluation of different portable dental X-ray units. Forensic Sci Int.

[11] Wenzel A (1994). Sensor noise in direct digital imaging (the RadioVisioGraphy, Sens-a-Ray, and Visualix/Vixa systems) evaluated by subtraction radiography. Oral Surg Oral Med Oral Pathol.

[12] Attaelmanan AG, Borg E, Grondahl HG (1999). Assessments of the physical performance of 2 generations of 2 direct digital intraoral sensors. Oral Surg Oral Med Oral Pathol Oral Radiol Endod.

[13] Faul F, Erdfelder E, Lang A-G, Buchner A (2007). G*Power 3: a flexible statistical power analysis program for the social, behavioral, and biomedical sciences. Behav Res Methods.

[14] Ulusu T, Bodur H, Odabas ME (2010). In vitro comparison of digital and conventional bitewing radiographs for the detection of approximal caries in primary teeth exposed and viewed by a new wireless handheld unit. Dento Maxillo Fac Radiol.

[15] Pittayapat P, Thevissen P, Fieuws S, Jacobs R, Willems G (2010). Forensic oral imaging quality of hand-held dental X-ray devices: comparison of two image receptors and two devices. Forensic Sci Int.

[16] Makdissi J, Pawar RR, Johnson B, Chong BS (2016). The effects of device position on the operator's radiation dose when using a handheld portable X-ray device. Dento Maxillo Fac Radiol.

[17] Rottke D, Gohlke L, Schrodel R, Hassfeld S, Schulze D (2018). Operator safety during the acquisition of intraoral images with a handheld and portable X-ray device. Dento Maxillo Fac Radiol.

